# Value of real-time endobronchial ultrasound-guided transbronchial needle aspiration

**DOI:** 10.4103/1817-1737.78422

**Published:** 2011

**Authors:** Erdogan Cetinkaya, Gulsah Gunluoglu, Akif Ozgul, Mehmet Z. Gunluoglu, Guler Ozgul, Ekrem C. Seyhan, Atayla Gencoglu, Sule Gul

**Affiliations:** Department of Chest Diseases, Yedikule Chest Diseases and Thoracic Surgery Education and Research Hospital, Istanbul, Turkey; 1 Department of Thoracic Surgery, Yedikule Chest Diseases and Thoracic Surgery Education and Research Hospital, Istanbul, Turkey

**Keywords:** Endobronchial ultrasound, lung cancer, mediastinum

## Abstract

**AIM:**

The diagnostic value of real-time convex-probe endobronchial ultrasound (CP-EBUS)-guided transbronchial needle aspiration (TBNA) in mediastinal pathologies, and the factors influencing it are not clearly known. This study has been designed to examine factors affecting the efficacy and diagnostic value of CP-EBUS-TBNA.

**METHODS:**

Patients (*n* = 321) with mediastinal mass or enlarged lymph node in the mediastinum, participated in this study, conducted between April 2007 and May 2009. Samples were obtained from the lesions using the TBNA method, with the guidance of CP-EBUS. Histopathologic (in all samples) and microbiologic (in 151 patients’ samples) evaluations were carried out. Biopsy using an appropriate surgical method was taken when the result of the TBNA procedure was nondiagnostic. Thirty-four patients were excluded from the analysis due to several reasons. The diagnostic efficacy of the procedure was analyzed in the remaining 287 patients.

**RESULTS:**

The diagnostic rate was 92% (89.7% for the benign diseases and 92.6% for the malignant diseases (*P* = 0.59)). In granulomatous diseases, the rate was 96% for sarcoidosis and 79% for tuberculosis (*P* = 0.002). Patients in whom only one lymph node was sampled and in whom two or more lymph nodes were sampled had a diagnostic rate of 85% and 95%, respectively (*P* = 0.03). When left hilar nodes were sampled, a higher diagnostic rate was achieved (*P* = 0.01).

**CONCLUSIONS:**

CP-EBUS-guided TBNA has a high diagnostic rate. Sampling of at least two separate lymph nodes and the discovery of left hilar station in these lymph nodes increase the rate of diagnosis.

Different techniques can be used for the diagnosis of diseases of mediastinal lymph nodes or masses. A frequently used method, bronchoscopy-guided TBNA, has a variable diagnostic success rate for hilar and mediastinal pathologies.[1] The combination of ultrasonography with flexible bronchoscopy (endobronchial ultrasound, EBUS) allows visualization of lesions in the mediastinum, lymph nodes, and vascular structures. Thus, the biopsy area can be targeted, and the diagnostic value of TBNA increases.[2] Initial EBUS procedures were done using radial probes.[3] Although these probes do not allow biopsies to be performed simultaneously, compared with conventional TBNA, they have increased the diagnostic efficacy especially of stations outside the subcarinal area.[4] Recently, the use of convex-probe endobronchial ultrasound (CP-EBUS) during EBUS enabled a biopsy to be conducted simultaneously with the visualization of the lesion in the mediastinum.[5]

In the present study, our goal was to examine factors affecting the efficacy and diagnostic value of CP-EBUS guided TBNA (EBUS-TBNA) in mediastinal and hilar pathologies.

## Methods

Between April 2007 and May 2009, EBUS-TBNA was conducted on 321 consecutive patients with noncystic masses and/or enlarged (short-axis diameter > 1 cm) lymph nodes in the mediastinum. This was a prospective study. All patients were informed of the procedure, and written consent was obtained. The ethics committee of our hospital approved the study (Decision number: 009-4-6).

Patients underwent the procedure after fasting for at least 4 h. Topical anesthesia was achieved using xylocaine (maximum 8 mg/kg), and conscious sedation was achieved using midazolam (0.05 mg/kg). Endobronchial ultrasonography was conducted using a fiberoptic ultrasound bronchoscope (CP-EBUS; BF-UC 160F-OL8; Olympus Medical Systems, Tokyo, Japan). The location, shape, and structure of the lesions were examined with ultrasound. The place of the stations was named and numbered using the lymph node map proposed by Mountain[6] During the EBUS examination in one patient, the lesion was seen as pulmonary artery dilatation. This observation was considered diagnostic, and the TBNA procedure was not conducted. The TBNA procedure was administered to all other patients. The bronchoscope was guided to the lesion, during real-time imaging, a 22-gauge biopsy needle (XNA-220), manufactured for this purpose, was pushed out from the distal tip of the bronchoscope and samples consisting of cells or tissue fragments were obtained. All procedures were conducted by a single bronchoscopist (EC). Because a pathologist was not present for rapid on-site evaluation (ROSE), it was not possible to evaluate whether the material contained enough cells. The bronchoscopist evaluated whether the procedure was sufficient for each sampled area.

In one patient, slight bleeding developed as a complication. The procedure was termed and not repeated in this patient. The bleeding stopped without operative intervention, but the material that had been obtained was found not enough for diagnosis. In 10 patients, a diagnosis could not be obtained from the initial EBUS-TBNA, and the same procedure was repeated; the records from both procedures were combined.

To conduct histopathologic examination, the materials were sent to the pathology department. If no indication of disease leading to a diagnosis was found, the sufficiency of the material obtained was evaluated. If mature and transformed lymphocyte colonies, histiocytes, anthracosis residue, macrophages, or histiocytes containing anthracosis were present, it was accepted that the lymph node was reached and the material was deemed to be sufficient; otherwise, it was assumed to be insufficient.

Although sufficient material was obtained, patients in whom a specific disease could not be identified or those who had cyto-pathological findings consistent with reactive hyperplastic lymph nodes were advised to have samples taken from the mediastinal lesions using an invasive procedure. Of these patients, 27 were excluded from the study because they did not provide consent; thus, disease presence could not be determined. Other patients who have a negative result after the procedure and who accepted the invasive diagnostic procedures, samples were again obtained from the mediastinum, by surgical methods (by mediastinoscopy in the most of the patients, and by thoracoscopy or thoracotomy in only five patients). Results obtained in this fashion were compared with TBNA results.

In seven patients, materials were insufficient. Final diagnoses which had been obtained after evaluation of surgical biopsies were granulomatous disease in three and, esophageal leiomyoma in one patient and no specific disease was determined in the other patients. These seven patients were excluded from the study.

When a specific diagnosis was reached from a sample, the procedure was considered diagnostic as long as no serious clinical inconsistency was found. In granulomatous disease, tuberculosis was diagnosed if an acid-fast bacilli was determined, wide necrosis was encountered, symptoms, tuberculosis contact history, and tuberculin skin test were positive/supportive, and a response to anti-tuberculosis treatment existed. Sarcoidosis was diagnosed if samples were non-necrotic, tuberculosis skin test or quantiferon blood test were negative, and the patient exhibited radiologically bilateral hilar enlargement, erythema nodosum, Löfgren’s syndrome, uveitis, and non-necrotisan granulomatous infection in skin nodules.

After the exclusion of 7 patients from whom enough material could not be obtained and 27 patients who refused to have the suggested invasive procedure performed to obtain a specific diagnosis, 287 patients made up the study group. One of these patients was supposed to have a sample taken during the EBUS procedure, but the lesion was found to be due to pulmonary artery dilatation; no samples were obtained from the patient. Despite this, the process was considered diagnostic, and the findings were added to the results from the 286 patients on whom the TBNA procedure was performed.

### Statistical analysis

Diagnostic success per patient was calculated. The rate of diagnostic accuracy, sensitivity, specificity, and positive and NPVs for the patients were calculated using standard formulations. Comparisons of these ratios in subgroups were performed using the MedCalc statistical software package (Version 11.1.1.0; MedCalc Software; Mariakerke, Belgium). Correlations were shown using the SPSS statistical software package (Statistical Package for Social Sciences (SPSS), version 10.0; SPSS, Inc.; Chicago, IL, USA), assisted by the Spearman test.

## Results

Of the 287 patients, 160 were male, 127 were female, and the average age was 50.2 (15–88) years. In total, 508 stations from 286 patients were sampled [1.8 (1–4) per patient]. A total of 1321 samples were obtained from these stations [4.6 (1–13) per patient and 2.6 per area]. The subcarinal station had the highest frequency of sampling (192 patients). This was followed by left hilar (*n* = 123), right lower paratracheal (*n* = 91), right hilar (*n* = 64), right upper paratracheal (*n* = 17), left lower paratracheal (*n* = 14), and left upper paratracheal (*n* = 1) stations. Other than slight bleeding in one patient, no other procedure-related complication was observed.

As a result of the EBUS-TBNA procedures, a diagnosis was achieved in 227 (79.1%) patients [Table 1]. Microbiologic evaluation of the TBNA biopsies showed acid-fast bacilli in only 5/151 patients. The diagnosis was confirmed as tuberculosis in these patients. To differentiate sarcoidosis from tuberculosis, criteria described in the “Material and Methods” section were used, in cyto-pathologic evaluation of the TBNA showed granulomatous disease patients. From 60 of the patients, no tissues with disease were obtained (negative result). In these patients, the diagnoses obtained from invasive methods are summarized in Table 2. Within this patient group, negative results were confirmed in 37 patients (61.7%); in 23 patients (38.3%), TBNA results were determined to be false negative. The diagnostic sensitivity of the EBUS-TBNA procedure was calculated to be 90.8%, and the negative predictive value (NPV) was 61.7%.

**Table 1:** Diagnoses obtained through the EBUS-TBNA biopsy method

Diagnosis	*N*
Granulomatous disease	139
Lymphoma	4
Lung cancer	79
Non-small cell lung cancer	64
Small cell lung cancer	15
Nonlung cancer	4
Pulmonary artery dilatation	1
Total	227
No evidence of disease	60
Grand total	287

**Table 2:** Diagnoses obtained after invasive procedures in patients who could not be diagnosed specifically as a result of EBUS-TBNA biopsy

Diagnosis	*n*
Benign diseases	16
Granulomatous disease	14
Benign mediastinal tumor (Ganglioneuroma)	1
Silicosis	1
Malignant diseases	7
Lung cancer	5
Non-lung cancer	1
Lymphoma	1
Total	23
No evidence of disease	37
Grand total	60

Based on each of the final diagnoses obtained, the diagnostic value of the EBUS-TBNA process was also calculated. Diagnostic rates can be found in Table 3. The general diagnostic rate was 92%, and no significant difference in diagnostic rates was found between benign and malignant diseases (89.7% and 92.6%, respectively, *P* = 0.59). When the success of the procedures for diagnosing sarcoidosis and tuberculosis were compared, the success rate was found to be significantly lower in tuberculosis patients (*P* = 0.002). The diagnostic rate in small cell cancer was 100%, and this rate did not differ significantly from the rate obtained from non-small cell cancer (*P* = 0.66).

**Table 3:** Diagnostic success of EBUS-TBNA biopsy according to each disease

Final diagnosis	Diagnosis obtained using EBUS-TBNA		
	*n*	*N*	*%*
Benign diseases	156	140	89.7
Granulomatous disease	153	139	90.8
Sarcoidosis	105	101	96.2
Tuberculosis	48	38	79.2
Other	3	2	66.6
Malignant disease	94	87	92.6
Lung cancer	84	79	94
Non-small cell lung cancer	69	64	92.8
Small cell lung cancer	15	15	100
Other	10	8	80
No evidence of disease	37	37	100
Total	287	264	92

**Table 4:** In patients who are diagnosed with a benign disease or no disease, the success of the EBUS-TBNA procedure based on the criteria whether some lymph nodes were sampled or not

Lymph node area	Sampling	*n*	Diagnosis		*P*
			*N*	*%*	
Right lower paratracheal (#4R)	Present	54	49	90.7
					0.99
	Absent	138	127	92
Subcarinal (#7)	Present	150	140	93.4
					0.19
	Absent	42	36	85.7
Right hilar (#10R)	Present	35	31	88.6
					0.69
	Absent	157	145	92.4
Left hilar (#10L)	Present	99	96	97
					0.01
	Absent	93	80	86

The number of samples obtained per mediastinal area was one, in one patient, and was more than two in all other patients. No significant correlation was found between the average number of samples obtained per each area and the diagnostic sufficiency (*r* = 0.1 and *P* = 0.1).

Once the patients with a final diagnosis of malignant disease and the patient from whom a sample could not be obtained due to pulmonary artery dilation were excluded, the effect of the number of sampled lymph node areas on diagnostic value was examined in the remaining 192 patients. In this series, average number of sampled mediastinal areas was two. Success of the procedure was compared between patients who had two or more areas sampled, and those who had only one area sampled. In 122 (95.3%) of 128 patients who had two or more lymph nodes sampled, a diagnosis could be reached, whereas only 54 (84.4%) of 64 patients who had only one lymph node sampled were diagnosed successfully. This difference was statistically significant (*P* = 0.02). In the same patient group, the effect of taking samples from various lymph nodes on the success of this procedure was analyzed. The ratios of patients who had successful procedures based on whether an area was sampled are summarized in Table 4. The diagnosis ratio was much higher in patients from whom a sample was obtained from the left hilar region compared with patients in whom this region was not sampled. On the other hand, in patients from whom a sample was obtained from the subcarinal region, although the diagnosis rate was higher compared with patients who were not sampled in this region, the difference was not statistically significant.

## Discussion

The TBNA procedure using a flexible bronchoscope has been used to obtain samples from hilar and mediastinal lymph nodes and masses for a long period of time.[7] However, a sufficient rate of success has been achieved by only sampling the subcarinal and right paratracheal areas[8–10] compared with other stations. For these reasons, the diagnostic success of this procedure has been unsatisfactory.[10] The limitations of the procedure have been partially overcome by the use of EBUS. However, initial EBUS applications using radial probe could only localize the sampled area using ultrasound visualization; samples were obtained blindly. Although this procedure increased the safety and efficacy of the biopsy, it remained unsatisfactory.[11] It was reported that the CP-EBUS-guided TBNA procedure had a high success rate in obtaining a diagnosis.[12] The high diagnostic rate obtained from our study confirms that this procedure is very effective. In addition to this, in almost all patients, the procedure was completed with no obvious complications. For this reason, EBUS-TBNA is also considered to be a very reliable procedure. The lack of need for general anesthesia is another advantage of the EBUS-TBNA procedure. In some published studies, it was preferred to conduct the procedure under general anesthesia. However, as in our study, local anesthesia and sedation are almost always sufficient.

In this study, we also aimed to analyze the factors that affected the probability of diagnoses made using EBUS-TBNA. In a review,[13] it was noted that the diagnosis of benign disease is affected by the pathologist’s experience, perhaps influencing the lower diagnostic success rate for benign disease. However, the high success rate for benign diseases obtained through our study shows the importance of CP-EBUS guidance. The lower diagnostic success rate determined for tuberculosis is not unexpected. For sarcoidosis, the diagnostic success rate of the EBUS-TBNA procedure was reported to be 85-91.8%[14–16] Compared to these rates, the diagnostic efficacy of conventional TBNA for tuberculosis is quite low.[17] The low diagnostic rate of tuberculous lymphadenitis may be due to the scarcity of acid-fast bacilli in the lymph node, and the scarcity of cellular material in the samples obtained from wide necrotic areas. However, 79% diagnostic rate obtained for tuberculosis in this study is quite high compared with the diagnostic success rate (65%) reported for TBNA.[17]

In lung cancer staging, three samples per lymph node station are reported to be sufficient.[18] However, no information has been published regarding the number of samples obtained per area from patients in whom procedures are conducted for diagnostic purposes. In our study, no correlation was found between an increased number of samples and successful diagnosis. This situation might indicate that CP-EBUS guidance guarantees a sufficient sample.

In our study, the success of the procedure per patient (not per lymph node) was analyzed. Thus, if a diagnosis was achieved based on a sample from any area, the procedure was considered successful. Malignant diseases do not have the tendency to involve all mediastinal areas. In these patients, sampling was directed to regions with a possibility of involvement based on thoracic CT, positron emission tomography, and CP-EBUS findings. Therefore, it seems unreasonable to analyze the success of the TBNA procedure based on whether a sample was obtained from a specific mediastinal region. On the contrary, granulomatous diseases generally involve the most of the mediastinal lymph nodes; as a result, one can analyze the sampling from different areas to determine which area would increase the success of the TBNA procedure. In our study, a high possibility of establishing a diagnosis was shown for patients from whom at least two mediastinal areas were sampled. Also, our study provides clues as to which lymph nodes should be chosen. In our analysis, a tendency was observed toward a higher rate of diagnosis for samples obtained from the subcarinal area compared with patients in whom samples from this area were not obtained; however, this higher rate did not reach a statistically significant level. Despite this, the probability of reaching a diagnosis was shown to be significantly high when the samples were obtained from the left hilar area. Based on the results of our study, we recommend sampling at least two regions during EBUS-TBNA in patients suspected to have granulomatous disease. We believe that one of the areas to be sampled should be the left hilar region. Granulomatous diseases typically involve hilar lymph nodes. Due to wideness of left hilar area, more lymph nodes can keep this location. These can be the causes of high success rate in diagnosing granulomatous diseases in left hilar area sampled patients. The subcarinal area should be considered when selecting other regions.

In conclusion, the EBUS-TBNA procedure is safe, and has a high diagnostic rate. The rate is relatively low in tuberculosis lymphadenitis. Even with a small number of samples, CP-EBUS guidance can help to obtain a diagnostic sample. For nonmalignant diseases, the probability of reaching a diagnosis is higher when at least two mediastinal regions including left hilar area are sampled.
